# Evaluation of the Potential Effect of Postbiotics Obtained from Honey Bees against *Varroa destructor* and Their Combination with Other Organic Products

**DOI:** 10.3390/insects15010067

**Published:** 2024-01-17

**Authors:** Eduardo José García-Vicente, María Benito-Murcia, María Martín, Ismael Rey-Casero, Ana Pérez, María González, Juan Manuel Alonso, David Risco

**Affiliations:** 1 Neobéitar S.L., Av. de Alemania, 6 1ºB, 10003 Cáceres, Spain; mariabenito@neobeitar.com (M.B.-M.); mariamartin@neobeitar.com (M.M.); ismaelrey@neobeitar.com (I.R.-C.); anaperez@neobeitar.com (A.P.); mariagonzalez@neobeitar.com (M.G.); 2 Department of Animal Medicine, Facultad de Veterinaria, Universidad de Extremadura, Av. de la Universidad s/n, 10001 Cáceres, Spain; riscope@unex.es; 3 Department of Animal Health, Facultad de Veterinaria, Universidad de Extremadura, Av. de la Universidad s/n, 10001 Cáceres, Spain; jmalonso@unex.es

**Keywords:** honey bee, *Varroa destructor*, lactic acid bacteria, postbiotic, oxalic acid, oregano essential oil, bioassay

## Abstract

**Simple Summary:**

*Varroa destructor* is a parasitic mite of honey bees that causes several injuries to colonies. The emergence of resistant populations of mites to traditional chemical acaricides has decreased their effectiveness and has made the control of this pathogen extremely difficult. The aim of this study was to evaluate the efficacy of postbiotics from beehives, and their combinations with other organic compounds, against *V. destructor*. Four species of lactic acid bacteria were obtained and tested by exposing mites to each species in a postbiotic format (preparations of inanimate microorganisms and/or their components that confer a health benefit to the host). Three species decreased the viability of mites regarding the control group, and those were further tested together as a single postbiotic product (POS). Later, the effect of the postbiotic product and its combination with oxalic acid and oregano essential oil was tested in eight groups: Control, POS, Oregano, Oxalic, POS/Oregano, POS/Oxalic, Oregano/Oxalic, POS/Oregano/Oxalic. All the products decreased the viability of the mites, and the most effective were the oxalic acid combinations, with significant differences for each product alone. These results show that oxalic acid combinations with postbiotics and essential oils could be an important tool for the treatment of *V. destructor*.

**Abstract:**

The *Varroa destructor* mite infests *Apis mellifera* colonies and causes significant harm. Traditional treatments have become less effective because of mite resistance development and can also generate residues inside beehives. This study aimed to gauge the efficacy of a beehive-derived postbiotic in reducing *V. destructor* viability and to explore its synergies with organic compounds. Four lactic acid bacteria (LAB) species, *Leuconostoc mesenteroides*, *Lactobacillus helsingborgensis*, *Bacillus velezensis*, and *Apilactobacillus kunkeei*, were isolated and tested in a postbiotic form (preparations of inanimate microorganisms and/or their components) via bioassays. *L. mesenteroides*, *L. helsingborgensis*, and *B. velezensis* notably reduced the mite viability compared to the control, and they were further tested together as a single postbiotic product (POS). Further bioassays were performed to assess the impact of the POS and its combinations with oxalic acid and oregano essential oil. The simple products and combinations (POS/Oregano, POS/Oxalic, Oregano/Oxalic, and POS/Oregano/Oxalic) decreased the mite viability. The most effective were the oxalic acid combinations (POS/Oregano/Oxalic, Oxalic/Oregano, POS/Oxalic), showing significant improvements compared to the individual products. These findings highlight the potential of combining organic products as a vital strategy for controlling *V. destructor* infection. This study suggests that these combinations could serve as essential tools for combating the impact of mites on bee colonies.

## 1. Introduction

*Varroa destructor*, a parasitic mite of honey bees (*Apis mellifera*), is considered one of the main risks for beekeeping worldwide. This species was described as *Varroa jacobsoni* until 2000, when different species of this genus were discovered by D.L. Anderson and J.W. Trueman [1]. Originally, it parasitized *Apis cerana* in Asia and then leaped to the European honey bee *A. mellifera* in the first half of the 20th century in Russia or the Far East, where both species converged because of beekeeping transhumant routes [2,3]. Subsequently, the mite spread quickly and has colonized beehives around almost all the world where humans manage honey bee colonies today, except some extreme northern territories and remote islands such as the Seychelles and Comoros archipelagoes [4,5,6].

Adult females of *V. destructor* parasitize adult bees, primarily feeding on their fat bodies and also consuming their hemolymph, while they remain attached to the bees; bodies [7]. They enter into the brood cells to breed immediately before they are sealed [5,8,9]. Infestations by *V. destructor* in a colony can cause several injuries to bees, such as improper development of wings, abdomen, legs or glands; malformations; weakness; and weight loss, among others [9,10,11]. Moreover, this mite can transmit many viruses, such as Acute Bee Paralysis Virus (ABPV), Israeli Acute Paralysis Virus (IAPV) and Deformed Wing Virus (DWV) [12,13,14,15], and act synergistically with them, causing serious damage to the colonies [16,17].

Traditional treatments for *V. destructor* include the application of chemical acaricides such as formamidines as amitraz, pyrethroids as tau-fluvalinate and flumethrin, or organophosphates as coumaphos [5]. Originally, these compounds were highly effective against the mites, but their effectiveness has decreased due to the appearance of resistant populations of mites by acquiring resistance genes or mechanisms against these molecules [18,19,20,21,22,23,24]. Moreover, these conventional acaricides generate residues that accumulate in wax, bee bread and honey, and they can migrate to brood and adult honey bees, with negative effects on the health of the colony [25,26,27,28,29,30].

In addition, organic substances such as oxalic acid, formic acid and thymol have also been used as treatments against *V. destructor*. The development of resistant populations due to the use of these compounds has not been described, but their use is not free from issues. Most of them have very narrow application temperature ranges, and outside of them, these compounds can greatly decrease their effectiveness or increase their toxic effects and the mortality of honey bees [31,32,33,34,35]. Other natural compounds, such as oregano essential oil, are being investigated as an alternative to control *V. destructor* in beehives, with very promising results under laboratory conditions [36].

Therefore, it is necessary to achieve the development of new natural products for the treatment of *V. destructor* that do not generate resistant populations of mites or toxic residues in the beehives or beekeeping products and that are safe for bees and beekeepers and easy to use.

Thus, bioactive compounds, mainly probiotics, have begun to be studied as natural compounds to control *V. destructor* infestation and improve honey bee health [37,38,39,40]. Although the mechanism of action is not entirely clear, it is thought to be related to factors such as the ability of bacteria to synthesize metabolites that inhibit the growth of pathogens, competition for nutrients, or stimulation of the immune system [41]. Some studies have investigated the beneficial effects of probiotics based on LABs against the mite under laboratory conditions [42,43]. De Piano et al. also studied in 2020 the effect of a postbiotic such as a cell-free supernatant obtained via culture of *Lactobacillus johnsonii* on the mortality of mites with promising results [44], but the effect of postbiotics from more LAB species, and their combination with other substances, have not been evaluated. Postbiotics are “preparations of inanimate microorganisms and/or their components that confer a health benefit on the host”. The word “inanimate” refers to the presence of live microorganism that have now been killed, without implying a loss of function, and preserving the metabolites and bacterial structures [45].

The aim of this study was to obtain lactic acid bacteria from the normal microbiome of honey bees and to evaluate their potential effects in a postbiotic format against *V. destructor* mites, alone or in combination with other organic compounds.

## 2. Materials and Methods

### 2.1. Bacteria Isolation

The samples were collected from four apiaries located in traditional beekeeping zones in Cáceres, Extremadura, Spain. The climatic conditions of this region are Mediterranean, with oceanic influence. Summers are warm, dry, and mostly clear, and winters are cold and partly cloudy. The vegetation that predominates in this zone is herbaceous vegetation, grassland, dryland cereal crops, stands of *Lavandula stoechas* and *Olea sylvestri*, *Populus alba* and *Salix* spp. on the banks. In addition, the pasture spaces of holm oak *Quercus rotundifolia* and cork oak *Quercus suber* stand out [46].

Apiaries were selected based on their low infestation by *V. destructor* levels according to records. In each apiary, one sample of approximately 30 adult bees and 25 cm^2^ of capped brood were taken from three different beehives randomly selected. In the laboratory, the brood was extracted from the cells and three pupae for the sample were placed in a Petri dish with solid selective for lactic acid bacteria (LAB) MRS culture media (Scharlau^®^, Barcelona, Spain), shaken for three minutes, and incubated for 48 h at 37 °C. Similarly, three adult bees were placed and cultured in MRS medium to obtain bacteria from their exoskeletons. Finally, the digestive tracts of 20 adult bees per sample were extracted and homogenized with five ml of sterile water in a stomacher bag for five minutes. Subsequently, one ml of the homogenized was inoculated into solid MRS culture media and incubated under the same conditions as previously described.

Different bacterial colonies obtained from the cultures were isolated in pure culture and identified via amplification of the 16S rRNA gene. DNA was extracted from each bacterial strain using a commercial kit (Patho Gene-spin^TM^ DNA/RNA Extraction Kit, iNtRON Biotechnology, Seongnam-si, Republic of Korea). Lastly, amplification of the fragment was performed using the DreamTaq Green PCR Master Mix (ThermoFisher Scientific^TM^, Waltham, MA, USA) and the conditions described in its data sheet [47], in a Biometra TOne 96 Series thermal cycler (Cultek S.L., Madrid, Spain). Sanger sequencing was performed with an ABI3730XL system (Applied Biosystems, Waltham, MA, USA) using PCR primers [48]. The sequences of the PCR products were compared with known 16S rRNA gene sequences in GenBank via multiple sequence alignment using MEGA11 Molecular Evolutionary Genetics Analysis version 11 software [49].

### 2.2. Postbiotic Elaboration

One strain of each bacterial species sequenced of honey bee microbiome was randomly selected to elaborate the different postbiotic products. Two colonies of each strain were inoculated in 50 mL of liquid MRS culture media (Scharlau^®^, Barcelona, Spain) and cultured at 37 °C for 48 h. The concentration of the growth obtained was measured by culturing serial dilutions. For that, 50 μL of each LAB growth serial dilution (from original to 10^−12^) was inoculated in solid MRS culture media and incubated at 37 °C for 48 h. After the incubation period, each colony of the cultures was counted, and thus the concentration of the growths was obtained. All the growths were adjusted to 10^9^ UFC/mL. Finally, the growths were inactivated using heat at 80 °C for 1 h to obtain them in a postbiotic format. The absence of live bacteria was studied by culturing them in the MRS medium.

### 2.3. LAB Bioassays

Beehive frames with a capped brood of colonies from commercial apiaries with high infestation by *V. destructor* (>10%) were taken to the laboratory. The brood cells were uncapped and the adult female mites were extracted with a brush and placed in an empty Petri dish of Ø100 mm in darkness and at room temperature, and they stayed there for 1 h at the most.

The base of one Petri dish of Ø55 mm for each LAB strain selected, and one more dish for the control group, were covered with filter paper impregnated with 150 μL of the appropriate LAB growth in the postbiotic format, or sterile MRS medium for the control. Twenty mites previously extracted were placed into each dish, sealed with Parafilm^®^ (Amcor, Zürich, Switzerland) and incubated in a double boiler at 34 °C for 6 h, as was previously described [19]. After the incubation period, the viability of the mites was evaluated and categorized as “0” or “dead”; “1” or “damaged”, when the mite showed incoordination, erratic movements, spasms or limited mobility; and “2” or “alive” (See Supplementary File S1). This was performed in triplicate for each strain, and only bioassays with mortality under 20% in the control group were considered. The mites were not fed during the period after extraction and before trials or in the incubation period.

### 2.4. Combined Bioassays

The LAB strains previously tested that showed effectiveness decreasing the viability of the mites were selected for the elaboration of a single product called the POS. The POS was prepared by adding the selected LAB strains in the postbiotic format in equal parts. To test the effectiveness of the combination of LABs, as well as its effect when it is combined with oregano essential oil (Oregano) and oxalic acid (Oxalic), eight different groups were established: Control, POS, Oregano, Oxalic, POS/Oregano, POS/Oxalic, Oregano/Oxalic, POS/Oregano/Oxalic.

The effectiveness of each product or combination of products on the viability of *V. destructor* was evaluated following the protocol described in the previous subchapter. In the groups that included oregano, the filter paper of the dishes was impregnated with 1 μL of a solution of oregano essential oil (0.125 mg/mL) and acetone, leaving the dish open for 15 min so that the acetone evaporated and only the essential oil remained on the filter paper. This concentration was chosen after checking that the concentration described in the bibliography [36] caused excessive mortality of the mites (unpublished data), and a minor effect was necessary to evaluate the combined organic products impact. For the rest of the groups, four solutions were produced: sterile MRS (for the control), POS (for POS and POS/Oregano), oxalic acid (40 mg/mL, using the same concentration of the commercial product Oxybee ^®^, Véto-pharma, France) in sterile MRS (for Oxalic and Oregano/Oxalic) and oxalic acid (40 mg/mL) in the POS (for POS/Oxalic and POS/Oregano/Oxalic), and the filter paper of each dish was impregnated with 150 μL of the pertinent solution. The protocols for the incubation and evaluation of mite viability were the same as those used previously (See Supplementary File S2). Similarly, they was performed in triplicate for each product and combination, and the mortality in the controls was always below 20%.

### 2.5. Data Analysis

The viability of the mites was compared among the groups in the LAB bioassays as well as in the combination bioassays. Parametric statistical tests for the comparison of means (one-way ANOVA (F)) were used when the variables showed a normal distribution. Otherwise, nonparametric statistical tests (Kruskal–Wallis test (H)) were performed, followed by post hoc tests (Tukey’s HSD test or pairwise Wilcoxon test), using the Benjamin–Hochberg (BH) adjustment method for multiple comparisons. Statistical analyses were performed using R v4.1.2 software. Differences were considered statistically significant when they were less than 0.05, and *p* values between 0.05 and 0.1 were considered marginally significant.

## 3. Results

A total of 25 bacterial colonies were isolated from the cultures. Six species were identified: *Leuconostoc mesenteroides*, *Staphylococcus epidermidis*, *Lactobacillus helsingborgensis*, *Staphylococcus warneri*, *Bacillus velezensis* and *Apilactobacillus kunkeei* (Table 1). Both species of the *Staphylococcus* genus were rejected from the experiment, and only lactic acid bacteria were selected for the bioassays.

Thus, the remaining four species of LAB were used in the postbiotic format to evaluate their effect on the viability of *V. destructor* mites. The mean viability of the mites from each group in the LAB bioassays is shown in Table 2. In the control group, the mean viability was 1.82, and three species of LAB, *L. mesenteroides* (H = 29.45, *p* = 1.4 × 10^−4^), *L. helsingborgensis* (H = 29.45, *p* = 6.9 × 10^−5^) and *B. velezensis* (H = 29.45, *p* = 5.3 × 10^−5^), decreased the mite viability significantly compared to the control. The decrease in viability was similar for the three species, and they did not show differences among them (H = 29.45, *p* = 1). However, the viability of the mites exposed to *A. kunkeei* was similar to the control and did not show significant differences (H = 29.45, *p* = 0.171).

**Figure 1:**
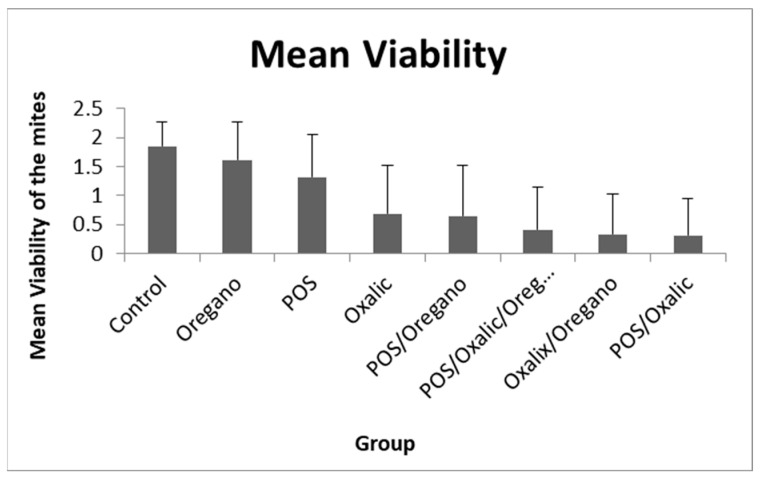
Mean viability of mites exposed to the different products and the control group. Top bars represent the standard deviation.

According to these results, *L. mesenteroides*, *B. velezensis* and *L. helsingborgensis* were selected and further tested together as a single postbiotic product (POS). The mean viability of the mites from each group in the combined bioassays is shown in Table 3 and Figure 1.

All the products tested showed a significant decrease in the viability of the mites regarding the control group. The single product with a higher efficacy in the mites was the oxalic acid (mean viability 0.68), followed by the POS (1.31), and lastly, the oregano essential oil (1.61); showing significant differences among them (see Table 4). The POS and Oregano combination decreased the viability (0.65) more than each one separately, and it was similar to the oxalic acid. The highest reduction in mite viability was produced by three different combined products: POS/Oxalic/Oregano (0.41), Oxalic/Oregano (0.33) and POS/Oxalic (0.31). All of them showed significant differences regarding the rest of the products and combinations, except for POS/Oxalic/Oregano with POS/Oregano, which showed marginally significant differences.

## 4. Discussion

The results showed that the postbiotic products derived from lactic acid bacteria isolated from the beehives decreased the viability of *V. destructor* mites, alone or in combination with other compounds, under laboratory conditions.

All the bacteria isolated from the intestinal tract and exoskeleton of adult bees and brood have been described as normal microorganisms in beehives [43,50,51,52,53,54]. The most abundant genera isolated from honey bee gut are *Staphylococcus*, *Enterococcus* and *Bacillus* [50], while the most isolated species from the honey bee surface is mostly *A. kunkeei*, followed by *Bacillus thuringiensis* [43]. The species obtained in this study belonged to the genera *Staphylococcus*, *Lactobacillus*, *Bacillus* and *Apilactobacillus*. The LABs species prevailed over other species due to the use of the specific culture media MRS. Finally, *L. mesenteroides* was the only species previously detected in pollen samples from beehives but not detected in honey bee samples [53]. Both species of the genus *Staphylococcus* were excluded from the bioassays owing to their pathogenic potential and their ability to transfer or acquire resistance or pathogenicity genes among different species or strains [55,56,57].

Regarding the lactic acid bacteria isolated in this study, only the effect of *A. kunkeei* against *V. destructor* and other honey bee pathogens such as *Nosema ceranae* or *Paenibacillus larvae* has been previously evaluated [43]. However, the remaining LAB species sourced from honey bee samples have been isolated but not yet evaluated as a potential method to manage this mite. Previous studies have primarily focused on other species within the same genus, particularly *Lactobacillus* (such as *Lactobacillus salivarius* and *Lactobacillus johnsonii*) and *Bacillus* (*Bacillus subtilis*) [40,42,44].

The results of the LAB bioassays showed that *L. helsingborgensis*, *B. velezensis* and *L. mesenteroides* in the postbiotic format had an effect against *V. destructor* regarding the control group, decreasing the viability of the mites. This effect is probably due to the production by these types of bacteria of organic acids such as lactic acid, phenyl lactic acid or acetic acid by these types of bacteria [42], since the negative effect of organic acids in *V. destructor* has been widely documented [35,58,59,60]. Other substances, such as metabolites and cell structures, may interfere with the mites too [41]. In contrast, although the mites exposed to *A. kunkeei* showed a certain trend toward lower viability, this group did not show significant differences with control group. These differences with results reported in previous studies [43] may be due to the need for a higher exposure time or dose to increase the mortality of the mites.

Relating to the combined bioassays results, to the best of our knowledge, there are no previous studies that have evaluated the possible synergistic effect of these different organic products.

The “simple” product with a higher effect was the oxalic acid, follow by the POS, and in last position, the oregano essential oil. The exposure of the mites to oxalic acid, and its very low pH, was the most effective treatment to reduce their viability. The postbiotic product also reduced the pH too via production of lactic acid during their culture [42], albeit to a lesser extent, although combined with the effect of their metabolites [41], this treatment also was effective against the mites. Moreover, the postbiotic product and oregano essential oil combination had a similar effect to oxalic acid, showing how the combination of the mechanism of action of postbiotics (production of lactic acid and secondary metabolites) and oregano (complex mixture of low molecular weight volatile substances such as hydrocarbons, oxygenated materials, phenylpropanoids and other compounds [36]) reaches an effect very similar to oxalic acid and is much higher than each compound separately. Finally, among the combined products, the oxalic acid combinations were most effective in decreasing mite viability: POS/Oxalic, Oxalic/Oregano and POS/Oxalic/Oregano, with no significant differences among them, but with a higher effect than oxalic acid alone. Thus, the great reduction in pH caused by oxalic acid combined with the action mechanisms previously described for the postbiotic, oregano essential oil or both was the best treatment to reduce the viability of *V. destructor* mites. These results could be the first step in finding new ways to effectively control *V. destructor* in beehives without using chemicals acaricides or other substances that can cause other detrimental effects. This supposes the first report of the effect of these combinations of natural products and could be the first step in finding new ways to effectively control of *V. destructor* in beehives without using chemicals acaricides or other substances that can cause other detrimental effects. Nevertheless, further research is needed to evaluate the effects of these compounds in commercial apiaries under field conditions, to check the security of products and possible application methods.

## Figures and Tables

**Table 1:** Number of isolated colonies, sample type and identification at the species level.

ID	Sample	Specie
1	Digestive tract	*Leuconostoc mesenteroides*
2	Adult bee exoskeleton	*Leuconostoc mesenteroides*
3	Brood exoskeleton	*Staphylococcus epidermidis*
4	Digestive tract	*Lactobacillus helsingborgensis*
5	Adult bee exoskeleton	*Staphylococcus warneri*
6	Brood exoskeleton	*Bacillus velezensis*
7	Brood exoskeleton	*Bacillus velezensis*
8	Brood exoskeleton	*Bacillus velezensis*
9	Digestive tract	*Staphylococcus epidermidis*
10	Digestive tract	*Lactobacillus helsingborgensis*
11	Adult bee exoskeleton	*Staphylococcus epidermidis*
12	Adult bee exoskeleton	*Lactobacillus helsingborgensis*
13	Adult bee exoskeleton	*Staphylococcus epidermidis*
14	Digestive tract	*Lactobacillus helsingborgensis*
15	Adult bee exoskeleton	*Lactobacillus helsingborgensis*
16	Digestive tract	*Lactobacillus helsingborgensis*
17	Digestive tract	*Lactobacillus helsingborgensis*
18	Adult bee exoskeleton	*Lactobacillus helsingborgensis*
19	Adult bee exoskeleton	*Lactobacillus helsingborgensis*
20	Digestive tract	*Lactobacillus helsingborgensis*
21	Digestive tract	*Lactobacillus helsingborgensis*
22	Adult bee exoskeleton	*Apilactobacillus kunkeei*
23	Adult bee exoskeleton	*Lactobacillus helsingborgensis*
24	Adult bee exoskeleton	*Apilactobacillus kunkeei*
25	Digestive tract	*Apilactobacillus kunkeei*

**Table 2:** Mean viability of mites ± standard deviation (sd) for the different species tested and control group.

LAB Species	Mean Viability ± sd
Control	1.82 ± 0.53
*L. mesenteroides* (LM)	1.48 ± 0.73
*A. kunkeei* (AK)	1.72 ± 0.6
*L. helsingborgensis* (LH)	1.54 ± 0.57
*B. velezensis* (BV)	1.52 ± 0.73

**Table 3:** Mean viability of mites ± standard deviation (sd) for the different products tested and control group.

Group	Mean Viability ± sd
Control	1.86 ± 0.4
POS	1.31 ± 0.74
Oxalic	0.68 ± 0.83
Oregano	1.61 ± 0.65
POS/Oxalic	0.31 ± 0.63
POS/Oregano	0.65 ± 0.87
Oxalic/Oregano	0.33 ± 0.7
POS/Oxalic/Oregano	0.41 ± 0.74

**Table 4:** *p* values of the pairwise Wilcoxon test post hoc comparison among the different products tested. Previous Kruskal–Wallis test H = 289.37. Cells colored with gray showed significant differences among the groups (*p* < 0.05). Different kinds of gray are used for better visual clarity of the results in function of *p* values magnitude (dark grey < 1 × 10^−8^; medium grey between 1.01 × 10^−8^ and 1 × 10^−3^; and light grey > 1.01 × 10^−3^).

	Control	Oregano	POS	Oxalic	POS/Oregano	POS/Oxalic/Oregano	Oxalic/Oregano	POS/Oxalic
Control	-	6.90 × 10^−4^	5.80 × 10^−10^	<2 × 10^−16^	<2 × 10^−16^	<2 × 10^−16^	<2 × 10^−16^	<2 × 10^−16^
Oregano	6.90 × 10^−4^	-	0.013	2.00 × 10^−9^	1.40 × 10^−10^	8.00 × 10^−16^	<2 × 10^−16^	6.30 × 10^−16^
POS	5.80 × 10^−10^	0.013	-	9.60 × 10^−5^	1.30 × 10^−5^	5.80 × 10^−10^	1.90 × 10^−11^	1.50 × 10^−10^
Oxalic	<2 × 10^−16^	2.00 × 10^−9^	9.60 × 10^−5^	-	0.701	0.035	0.006	0.011
POS/Oregano	<2 × 10^−16^	1.40 × 10^−10^	1.30 × 10^−5^	0.701	-	0.085	0.016	0.026
POS/Oxalic/Oregano	<2 × 10^−16^	8.00 × 10^−16^	5.80 × 10^−10^	0.035	0.085	-	0.477	0.554
Oxalic/Oregano	<2 × 10^−16^	<2 × 10^−16^	1.90 × 10^−11^	0.006	0.016	0.477	-	0.914
POS/Oxalic	<2 × 10^−16^	6.30 × 10^−16^	1.50 × 10^−10^	0.011	0.026	0.554	0.914	-

## Data Availability

The data presented in this study are available in Figure 1, Supplementary File S1 and Supplementary File S2.

## Supplementary Materials

The following supporting information can be downloaded at https://www.mdpi.com/article/10.3390/insects15010067/s1, Supplementary File S1: LAB Bioassays Data; Supplementary File S2: Combined Bioassays Data.
